# Supplementary material for: A perfusion-independent high-throughput method to isolate liver sinusoidal endothelial cells
Source: Commun Biol. 2025 Jan 8;8:22. doi: 10.1038/s42003-025-07458-5 (PMC11711496; doi:10.1038/s42003-025-07458-5)
Supplement: Supplementary file 2 — Supplementary Information [file 42003_2025_7458_MOESM2_ESM.pdf]

A perfusion-independent high-throughput method to isolate liver sinusoidal endothelial cells

Anna Babin-Ebell Gonçalves[1,2,#], Yifang Mao[1,3,12,#], Tinja Baljkas[4,5,#], Felix Wiedmann[6,7], Larissa Eis[8], Franziska Pilz[1], Manuel Winkler[4,5], Sina W. Kürschner-Zacharias[4,5], Marlene Hoffarth[1,12], Charlotta Funaya[8], Réza Shahidi[8], Cyrill Géraud[4,9,10], Chi-Chung Wu[11,12], Constanze Schmidt[6,7,12], Sergij Goerdt[4,5], Philipp-Sebastian Reiners-Koch[4,5], Mahak Singhal[1,7,12]

Supplemental Figures

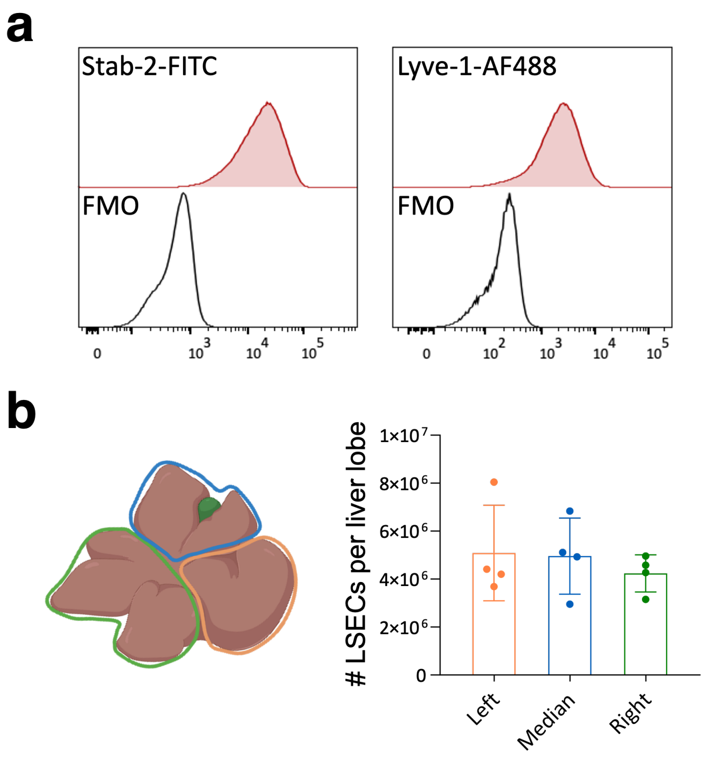

Supplementary Figure S1. Validating LSECs and isolating them from different lobes of the liver. (**a**) Liver endothelial cells isolated with perfusion-independent digestion method were stained for Stabilin-2 and Lyve-1 and analyzed with flow cytometry. Fluorescence minus one (FMO) stained samples served as corresponding controls. (**b, left**) The liver tissue was divided into three parts as shown in the picture. (**b, right**) Each part was processed to isolate LSECs. The dot plot shows the total count of LSECs isolated from each part of the liver [mean ± SD, n = 4 mice].

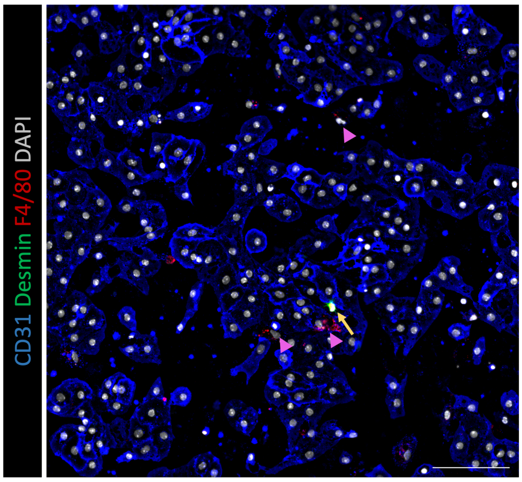

Supplementary Figure S2. Analyzing the primary LSEC cultures for contaminations. The image shows primary LSEC culture stained with for an endothelial cell-specific (CD31, in blue), stellate cell-specific (Desmin, in green), macrophage-specific (F4/80, in red), and nuclear marker (DAPI, in grey). Pink arrowheads highlight F4/80-positive macrophages, and the yellow arrow indicates a Desmin-positive stellate cell. Scale bar = 100 µm.

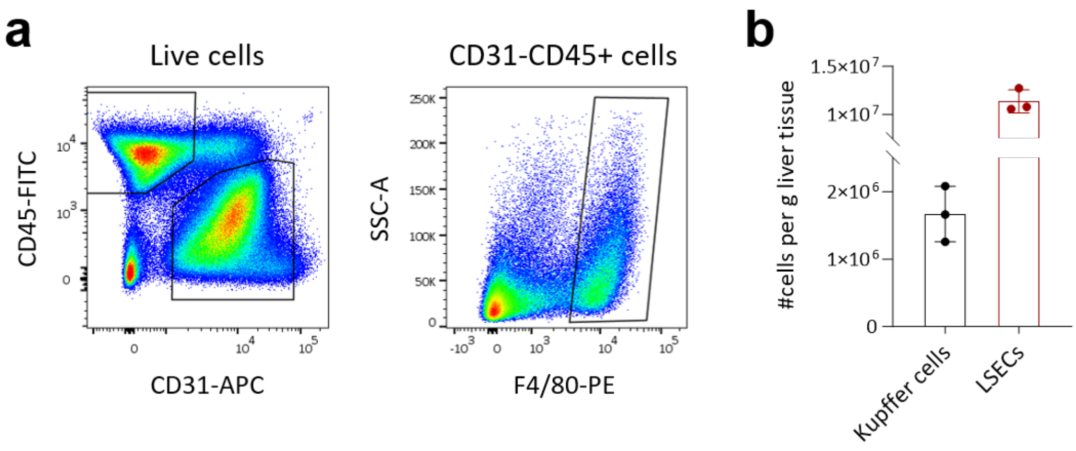

Supplementary Figure S3. Isolating Kupffer cells and LSECs from the liver. (**a**) FACS gating strategy to analyze and sort LSECs and Kupffer cells. All live cells were stratified based on the expression of CD45 and CD31. CD31+ cells were gated as LSECs. CD31-CD45+ cells were further analyzed for the expression of F4/80. CD31-CD45+F4/80+ cells were isolated as Kupffer cells. (**b**) The dot plot shows the number of isolated LSECs and Kupffer cells from the liver. [mean ± SD, n = 3 mice].
