# Supplementary material for: A perfusion-independent high-throughput method to isolate liver sinusoidal endothelial cells
Source: Commun Biol. 2025 Jan 8;8:22. doi: 10.1038/s42003-025-07458-5 (PMC11711496; doi:10.1038/s42003-025-07458-5)
Supplement: Supplementary file 4 — Description of Additional Supplementary Files [file 42003_2025_7458_MOESM4_ESM.docx]

Description of Additional Supplementary Files

**File name:** Supplementary Data

**Description:** The source data for all graphs in the manuscript figures.
