# Supplementary material for: A perfusion-independent high-throughput method to isolate liver sinusoidal endothelial cells
Source: Commun Biol. 2025 Jan 8;8:22. doi: 10.1038/s42003-025-07458-5 (PMC11711496; doi:10.1038/s42003-025-07458-5)
Supplement: Supplementary file 5 — reporting-summary [file 42003_2025_7458_MOESM5_ESM.pdf]

nature portfolio

Corresponding author(s): Mahak Singhal

Last updated by author(s): Dec 19, 2024

Reporting Summary

Nature Portfolio wishes to improve the reproducibility of the work that we publish. This form provides structure for consistency and transparency in reporting. For further information on Nature Portfolio policies, see our Editorial Policies and the Editorial Policy Checklist.

Statistics

For all statistical analyses, confirm that the following items are present in the figure legend, table legend, main text, or Methods section.

n/a	Confirmed	
☐	☒	The exact sample size (*n*) for each experimental group/condition, given as a discrete number and unit of measurement
☐	☒	A statement on whether measurements were taken from distinct samples or whether the same sample was measured repeatedly
☐	☒	The statistical test(s) used AND whether they are one- or two-sided<br>*Only common tests should be described solely by name; describe more complex techniques in the Methods section.*
☒	☐	A description of all covariates tested
☐	☒	A description of any assumptions or corrections, such as tests of normality and adjustment for multiple comparisons
☐	☒	A full description of the statistical parameters including central tendency (e.g. means) or other basic estimates (e.g. regression coefficient) AND variation (e.g. standard deviation) or associated estimates of uncertainty (e.g. confidence intervals)
☐	☒	For null hypothesis testing, the test statistic (e.g. *F*, *t*, *r*) with confidence intervals, effect sizes, degrees of freedom and *P* value noted<br>*Give P values as exact values whenever suitable.*
☒	☐	For Bayesian analysis, information on the choice of priors and Markov chain Monte Carlo settings
☒	☐	For hierarchical and complex designs, identification of the appropriate level for tests and full reporting of outcomes
☒	☐	Estimates of effect sizes (e.g. Cohen's *d*, Pearson's *r*), indicating how they were calculated

Our web collection on statistics for biologists contains articles on many of the points above.

Software and code

Policy information about availability of computer code

Data collection	QPCR analyses were performed with QuantStudio 5 system (Thermo Scientific). Liver histology images were acquired using a Zeiss AxioScan slide scanner. Immunofluorescence images were acquired using a Leica Thunder imaging platform.
Data analysis	All data analyses were performed with commonly used software and stated in the Methods section. In brief, qPCR data were analysed with QuantStudio 5 qPCR data analysis software. Flow cytometry data were analysed with FlowJo software. GraphPad Prism 10 was used for statistical analysis. Fiji was used for image analysis.

For manuscripts utilizing custom algorithms or software that are central to the research but not yet described in published literature, software must be made available to editors and reviewers. We strongly encourage code deposition in a community repository (e.g. GitHub). See the Nature Portfolio guidelines for submitting code & software for further information.

Data

Policy information about availability of data

All manuscripts must include a data availability statement. This statement should provide the following information, where applicable:

- Accession codes, unique identifiers, or web links for publicly available datasets
- A description of any restrictions on data availability
- For clinical datasets or third party data, please ensure that the statement adheres to our policy

There were no new data sets generated in this study.

Research involving human participants, their data, or biological material

Policy information about studies with human participants or human data. See also policy information about sex, gender (identity/presentation), and sexual orientation and race, ethnicity and racism.

Reporting on sex and gender	NA
Reporting on race, ethnicity, or other socially relevant groupings	NA
Population characteristics	NA
Recruitment	NA
Ethics oversight	NA

Note that full information on the approval of the study protocol must also be provided in the manuscript.

Field-specific reporting

Please select the one below that is the best fit for your research. If you are not sure, read the appropriate sections before making your selection.

☒ Life sciences ☐ Behavioural & social sciences ☐ Ecological, evolutionary & environmental sciences

For a reference copy of the document with all sections, see nature.com/documents/nr-reporting-summary-flat.pdf

Life sciences study design

All studies must disclose on these points even when the disclosure is negative.

Sample size	This was a hypothesis-driven study with unknown "effect magnitude". Therefore, statistical power was not computed prior to the experiments. Employed sample sizes were estimated based on the previous experiences with the experimental models.
Data exclusions	No data were excluded.
Replication	Individual mice served as biological replicates within an in vivo experiment.
Randomization	No randomization was performed.
Blinding	Analyses were performed by scientists without the knowledge of biological groups.

Reporting for specific materials, systems and methods

We require information from authors about some types of materials, experimental systems and methods used in many studies. Here, indicate whether each material, system or method listed is relevant to your study. If you are not sure if a list item applies to your research, read the appropriate section before selecting a response.

Materials & experimental systems

n/a	Involved in the study	
☐	☒	Antibodies
☒	☐	Eukaryotic cell lines
☒	☐	Palaeontology and archaeology
☐	☒	Animals and other organisms
☒	☐	Clinical data
☒	☐	Dual use research of concern
☒	☐	Plants

Methods

n/a	Involved in the study	
☒	☐	ChIP-seq
☐	☒	Flow cytometry
☒	☐	MRI-based neuroimaging

Antibodies

Antibodies used	For flow cytometry analysis, following antibodies were used anti-mouse CD45 (30-F11), anti-mouse CD31 (MEC 13.3), anti-mouse CD146 (ME9F1), anti-mouse CD117 (2B8), anti-mouse LYVE1 (ALY7), anti-mouse F4/80 (BM8), anti-mouse STAB2 (BS-12346R-FITC), and anti-pig CD31 [Cat #MCA1746APC]. We have provided a table with all fluorophore conjugations and catalouge numbers in the

	supplementary materials.<br><br>For IF staining, following antibodies were used – primary {goat anti-mouse CD31 [R&D Systems, Cat #AF3628], goat anti-mouse CD32b [R&D Systems, Cat #AF1460], rabbit anti-mouse Desmin [Abcam, Cat # ab15200], rabbit anti-mouse ERG [Abcam, Cat #ab196149], rat anti-mouse F4/80 [BioLeegend, # 123102], and rabbit anti-mouse Stabilin-2 [provided by Prof. Cyrill Géraud]}, and secondary {AF488-conjugated donkey anti-goat [Thermo Fisher Scientific, Catalog # A-11055], AF647-conjugated donkey anti-rabbit [Thermo Fisher Scientific, Cat #A-31573], AF555-conjugated donkey anti-goat [Thermo Fisher Scientific, Cat #A-32816], and AF488-conjugated donkey anti-rat [Thermo Fisher Scientific, Cat #A-21208]} antibodies.
Validation	All antibodies were used according to the manufacturers' instructions.

Animals and other research organisms

Policy information about studies involving animals; ARRIVE guidelines recommended for reporting animal research, and Sex and Gender in Research

Laboratory animals	Mice were purchased from Janvier.
Wild animals	NA
Reporting on sex	We used mixed female and male mice.
Field-collected samples	NA
Ethics oversight	All animal experiments were approved by the governmental and institutional Animal Care and Use Committees. All experiments were performed in accordance with the respective institutional guidelines for the care and use of laboratory animals.

Note that full information on the approval of the study protocol must also be provided in the manuscript.

Plants

Seed stocks	NA
Novel plant genotypes	NA
Authentication	NA

Flow Cytometry

Plots

Confirm that:

☒ The axis labels state the marker and fluorochrome used (e.g. CD4-FITC).

☒ The axis scales are clearly visible. Include numbers along axes only for bottom left plot of group (a 'group' is an analysis of identical markers).

☒ All plots are contour plots with outliers or pseudocolor plots.

☒ A numerical value for number of cells or percentage (with statistics) is provided.

Methodology

Sample preparation	Tissues were dissociated into single cell suspension with Liberase digestion enzyme mix (Roche). Following that, LSECs were enriched using CD146 microbeads (Miltenyi Biotec) according to the manufacturer's instructions.
Instrument	Analysis and cell sorting were carried out on BD Aria II or Melody cell sorting platform.
Software	Data were analysed with FlowJo software (Tree Star).
Cell population abundance	A small fraction of the sorted cells was re-analysed to assess the purity of isolated cells. Purity was found >95% in all analysed samples.
Gating strategy	For all analysed samples, cells were first gated for FSC-A/SSC-A to exclude debris. Secondly, single cells were selected based

Gating strategy

on the SSC-A/SSC-H gate. Thirdly, dead cells were excluded with FxCycle™ Violet staining. Afterward, live single cells were applied to the gates previously set up based on single stained samples.

☒ Tick this box to confirm that a figure exemplifying the gating strategy is provided in the Supplementary Information.
