# Supplementary material for: A perfusion-independent high-throughput method to isolate liver sinusoidal endothelial cells
Source: Commun Biol. 2025 Jan 8;8:22. doi: 10.1038/s42003-025-07458-5 (PMC11711496; doi:10.1038/s42003-025-07458-5)
Supplement: Supplementary file 1 — Transparent Peer Review file [file 42003_2025_7458_MOESM1_ESM.pdf]

nature portfolio

Peer Review File

A perfusion-independent high-throughput method to isolate liver sinusoidal endothelial cells

Corresponding Author: Dr Mahak Singhal

This file contains all reviewer reports in order by version, followed by all author rebuttals in order by version.

Version 0:

Reviewer comments:

Reviewer #1

(Remarks to the Author)
The work by Gonçalves et al. presents a novel protocol for isolating sinusoidal endothelial cells (LSEC) from mouse livers, with the approach further applied to fibrotic mouse livers and pig livers, with the aim to demonstrate its broader applicability. The protocol utilizes a perfusion-independent, ex vivo liver digestion method, which involves the mechanical disruption (chopping) and enzymatic digestion of the liver, followed by a two-step enrichment. Step II involves the enrichment of non-parenchymal cells (NPCs) via magnetic-bead-based positive selection using anti-CD146, while Step III achieves the isolation of high-purity LSECs through FACS sorting of CD31/CD146-positive cells.
Although this method results in a higher yield of LSECs from mouse livers (compared to numbers from liver perfusion-dependent methods, however in my view, it does not achieve high yields from fibrotic livers or pig livers. The authors should further discuss the limitations of a "dual sorting technique" (magnetic beads and FACS) in applications other than gene expression analysis (in terms of viable numbers of LSEC retrieved)

Several key aspects of the protocol, especially related to Step III (isolation of high-purity LSECs), require further explanations:

Point 1: CD146-Based Selection (Figure 2a):
The use of magnetic beads targeting CD146 restricts this protocol to mouse LSECs, as CD146 is not expressed on LSECs in humans and pigs; as in Figure 3d, only CD31-positive cell selection is shown (if observed, it should be stated). FACS analysis following CD146-positive selection indicates a significant number of viable CD45-positive cells.
Could the authors provide additional information on the murine CD146-positive liver cell population, considering that CD146 is also expressed on immune cells, liver NK cells, and stellate cells (pericytes)? Furthermore, could the authors comment on the CD45-negative cell population that is also negative for both CD146 and CD31? What types of cells are these?
Point 2: Clarification of LSEC Numbers and FACS Analysis (Figure 2):
The figure legend for Figure 2 should clearly describe what the dot plot represents. Are the LSEC numbers evaluated by FACS analysis reflective of sorted cell numbers using fluorescent FACS beads? How many sorted CD31/CD146-positive cells were used for the gene expression analysis presented in Figure 3c? Additionally, do the sorted cells remain viable in culture? Please include the scale values for the size bars in Figure 2c to improve clarity.
LSEC numbers are evaluated by FACS analysis (evaluated using fluorescent FACS beads) and are reflecting numbers of sorted cells? How many sorted CD31/CD146 positive cells have been used for gene expression analysis described in Figure 3c? Do the sorted cells survive in culture?

Point 3: Controversy Regarding CD31 as an LSEC Marker
CD31 expression in mouse LSECs has been a subject of debate, as it is sometimes associated with LSEC capillarization or endothelial cells from central veins. To address this, the authors should provide the percentage of Stabilin2-positive and Stabilin2-negative cells within the CD31/CD146 population, as determined by FACS analysis.

Point 4: Heterogeneity of Cultured Cells (Figure 4a)
In Figure 4a, the CD146-positive cells sorted by magnetic beads and stained with CD31 and CD32b do not exhibit membrane-specific staining, and the adherent cell population appears highly heterogeneous, as also seen in the FACS analysis and brightfield image. Has additional staining been performed to evaluate the presence of macrophages or stellate cells within the adherent population?

Point 5: Comparative analysis with perfusion-based isolation
One advantage of perfusion-based LSEC isolation followed by a density gradient for NPCs is the simultaneous isolation of both LSECs and macrophages. Did the authors evaluate the viability and number of macrophages within the CD146-negative (flow-through) population? Does this new digestion protocol enable efficient isolation of viable macrophages using anti-CD11b or F4/80 magnetic beads? This question is relevant given the suggestions in the discussion about the protocol's potential broader use.

Point 6: Use of CD117 as a marker for zonation
CD117 (c-Kit) is primarily known for its role in hematopoiesis, stem cell maintenance, and tissue regeneration and is more commonly associated with hepatic progenitor cells or stellate cells. Its use as a marker for LSEC zonation is less established.
Could the authors explain their rationale for using CD117 in this context? Also, the methodology for CD117-based stratification should be more detailed. Were different CD117-expressing populations sorted from a double-stained CD146/CD31 sorted population (is this the sequential approach?)
The total number of CD117-high, CD117-mid, and CD117-low sorted cells should also be expressed per gram of liver tissue.
Additionally, please specify the fluorescent-labeled antibodies used, not only the antibody clones.

Also, why wasn't LYVE1, a more traditional marker for LSEC zonation, used for stratification? What was the LYVE1 gene expression in the CD117-sorted cells, as shown in Figure 4d?

In the introduction, the statement "The accumulation of excessive extracellular matrix requires longer perfusion times with digestion mix and negatively affects the yield of LSEC isolation" needs a reference.

Reviewer #2

(Remarks to the Author)
Referee report:
A perfusion-independent high-throughput method to isolate liver sinusoidal endothelial cells.

The authors present a perfusion-independent protocol for isolation and purification of mouse liver sinusoidal endothelial cells (LSECs), where they chop the liver tissue and digest the tissue with liberase solution followed by differential centrifugation to remove hepatocytes. They then use a combination of MACS (CD31+,CD146+), and FACS (sorting viable, CD45- CD31+ cells) for cell purification. Liberase is commonly used in perfusion-based methods for LSEC isolation, and known to be effective while at the same time gentle to the cells. Cell yield per gram liver in the presented method is comparable to established perfusion-dependent methods which represents an improvement compared to other perfusion-independent protocols for LSEC isolation. The method further allows isolation of LSECs from both normal and fibrotic liver samples. A simplified method was used for purification of pig LSECs.

I find the protocol well described and potentially very useful. My specific comments to the study are linked to the characterization of the purified cells.

1. The endothelial markers CD31 and/or CD146 do not distinguish between LSEC and other endothelial cells in liver, although LSECs represent by far the largest population of liver endothelial cells. A morphological hallmark of LSECs is their numerous fenestrae, which are open nanosized holes with a diameter below the resolution limit of the light microscope (average diameter 100-200 nm). The purified endothelial cells from mouse and pig liver should therefore be characterized by electron microscopy or other super resolution imaging methods which allow for visualization of fenestrations, as part of the phenotyping of the cells in this method paper.
2. Have you observed any differences in plating efficiency, spreading and survival in culture of cells that has been through both MACS and FACS purification steps, versus MACS alone? This is of interest for the choice of purification method when performing functional studies in primary LSEC cultures.

Reviewer #3

(Remarks to the Author)
This is an experimental study, describing a novel strategy to isolate liver sinusoidal endothelial cells (LSECs) from mice and pigs.
LSECs are notriously difficult to isolate and culture and therefore, any protocol to improve isolation and culture of LSECs is welcome.
The method described in this study is a significant improvement over existing methods, especially with respect to ease of isolation, time required and yield and will make it possible for many more groups to study this interesting, but difficult to obtain, cell type.

However, what is lacking are functional studies with the LSECs that are obtained, as well as a more detailed characterisation.
E.g.:
1) How long can these LSECs be kept in culture in a differentiated state; does it differ from perfusion-dependent obtained LSCEs?
2) Do these LSECs have fenestrae, confirmed by electron microscopy?
3) The authors rely heavily on CD31 as marker for LSECs. LSECs have been reported to specifically express also other LSECs specific markers, e.g. LYVE-1, stabilins (as reported previously by this group), scavenger receptors, eNOS (not specific for LSECs, but a functional trait of all endothelial cells.

4) How did the authors rule out the contamination of non-sinusoidal endothelial cells?

Version 1:

Reviewer comments:

Reviewer #1

(Remarks to the Author)

The revised manuscript has been significantly improved, and my previous concerns and questions have been addressed thoroughly.

Reviewer #2

(Remarks to the Author)
The authors have very nicely answered all my questions.

Reviewer #3

(Remarks to the Author)
The authors significantly improved the quality of the manuscript and addressed all my comments and concerns in a satisfactory way.

Open Access This Peer Review File is licensed under a Creative Commons Attribution 4.0 International License, which permits use, sharing, adaptation, distribution and reproduction in any medium or format, as long as you give appropriate credit to the original author(s) and the source, provide a link to the Creative Commons license, and indicate if changes were made.

In cases where reviewers are anonymous, credit should be given to 'Anonymous Referee' and the source.
The images or other third party material in this Peer Review File are included in the article’s Creative Commons license, unless indicated otherwise in a credit line to the material. If material is not included in the article’s Creative Commons license and your intended use is not permitted by statutory regulation or exceeds the permitted use, you will need to obtain permission directly from the copyright holder.
To view a copy of this license, visit https://creativecommons.org/licenses/by/4.0/

Response to Reviewer's comments

Reviewer #1

GENERAL COMMENT: The work by Gonçalves et al. presents a novel protocol for isolating sinusoidal endothelial cells (LSEC) from healthy mouse livers, with the approach further applied to fibrotic mouse livers and healthy pig livers, with the aim to demonstrate its broader applicability. The protocol utilizes a perfusion-independent, ex vivo liver digestion method, which involves the mechanical disruption (chopping) and enzymatic digestion of the liver, followed by a two-step enrichment. Step II involves the enrichment of non-parenchymal cells (NPCs) via magnetic-bead-based positive selection using anti-CD146, while Step III achieves the isolation of high-purity LSECs through FACS sorting of CD31/CD146-positive cells.

Although this method results in a higher yield of LSECs from healthy mouse livers (compared to numbers from liver perfusion-dependent methods, however in my view, it does not achieve high yields from fibrotic livers or pig livers. The authors should further discuss the limitations of a "dual sorting technique" (magnetic beads and FACS) in applications other than gene expression analysis (in terms of viable numbers of LSEC retrieved)

Several key aspects of the protocol, especially related to Step III (isolation of high-purity LSECs), require further explanations:

RESPONSE TO GENERAL COMMENT: We sincerely thank the reviewer for his/her critical assessment and constructive feedback on our manuscript. A whole-hearted effort was made to address all comments of the reviewer which – we believe – has advanced our manuscript further.

Concerning the yield of LSECs with our isolation method, we obtained 10 x 10^6 LSECs per gram of healthy adult mouse liver tissue. For the pig liver, we obtained an average of 20 x 10^6 LSECs per gram of pig liver tissue, translating into 1.5 x 10^{10} LSECs per adult Landrace pig liver (weighing ~750g). These LSEC numbers from pig tissues are even higher compared to previous publications, where the authors obtained 2.8 x 10^9 sinusoidal cells per adolescent Norwegian pig liver (weighing ~237g) using perfusion-based digestion of liver tissues (1,2).

Regarding the fibrotic liver tissues, we agree with the reviewer's observation that the yield is lower, compared to the LSEC yield from healthy liver tissues, with an average of 1.7 x 10^6 LSECs per gram of mouse fibrotic liver tissue, translating into 3.4 x 10^6 LSECs per mouse fibrotic liver (weighing ~2g). Yet, these numbers are similar to a recent publication describing an optimized perfusion-dependent digestion procedure for isolating LSECs from fibrotic livers where the authors obtained 2-5 x 10^6 LSECs per mouse fibrotic liver (3).

Overall, our method is at least non-inferior, if not superior, to current perfusion-dependent liver digestion procedures for isolating LSECs. At the same time, our method offers a high throughput where multiple liver tissue samples can be processed at the same time for downstream analyses. We have discussed the limitations of the dual sorting approach in the revised manuscript.

COMMENT 1: CD146-Based Selection (Figure 2a): The use of magnetic beads targeting CD146 restricts this protocol to mouse LSECs, as CD146 is not expressed on LSECs in humans and pigs; as in Figure 3d, only CD31-positive cell selection is shown (if observed, it should be stated). FACS analysis following CD146-positive selection indicates a significant number of viable CD45-positive cells.

Could the authors provide additional information on the murine CD146-positive liver cell population, considering that CD146 is also expressed on immune cells, liver NK cells, and stellate cells (pericytes)? Furthermore, could the authors comment on the CD45-negative cell population that is also negative for both CD146 and CD31? What types of cells are these?

RESPONSE 1: We appreciate the reviewer's thoroughness and raising a critical point about the expression of CD146. In the current study, we employed a magnetic CD146-positive selection for enriching LSECs from non-parenchymal cell (NPC) suspension of mouse liver tissues only. We intentionally opted for this procedure as this is very much in line with current literature where the majority of perfusion-dependent protocols subsequently employ the CD146-based magnetic selection for isolating LSECs. This would allow wider acceptance of our perfusion-independent digestion method for the liver research community working with mice as an experimental model. In mouse liver, CD146 (Mcam) is primarily expressed in LSECs among different liver cell types as shown in the **Response Figure 1a** (showing the reanalysis of the Tabula Muris liver dataset). Additionally, further characterization of our primary cultures of LSECs revealed that the majority (nearly all) of CD146-enriched cells were positively stained for CD31 and ERG (endothelial cell-specific markers). Yet, there are 1-2 non-endothelial cells per field of view corresponding to <2% of all DAPI+ cells (**Fig. 4a** of the revised manuscript). Further characterization found that these contaminating cells are positive for either Des (stellate cells) or F4/80 (macrophages) [**Fig. S2** of the revised manuscript].

For isolating endothelial cells from pig liver tissues, we directly stained the NPC suspension with fluorophore-conjugated CD31 antibody and employed a FACS-based isolation of pig liver endothelial cells as shown in **Fig. 3d**. We didn't pursue any magnetic selection as there are currently no positive selection microbeads available for pig CD31.

As shown in **Response Figure 1b**, we plotted the mentioned triple-negative population (CD45-CD31-CD146-) on FSC-A/SSC-A and observed that these are quite small sized events. Given that they are negative for all analyzed markers and very small in size, we believe these events are primarily debris.

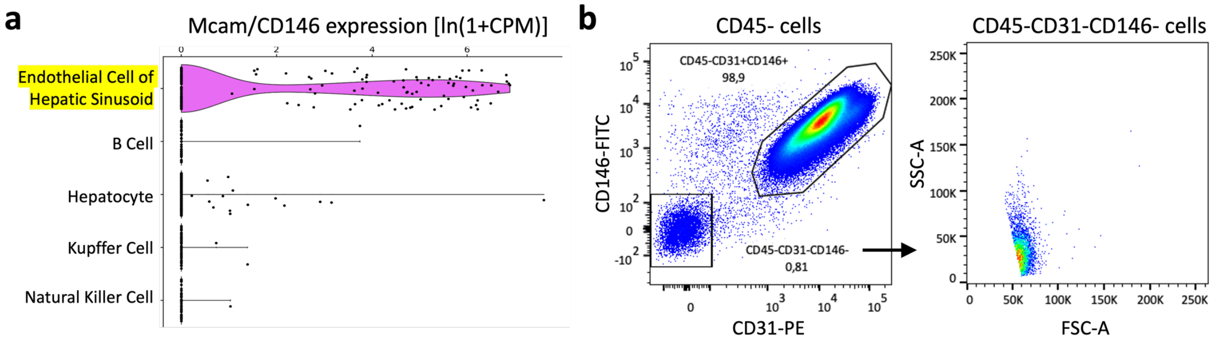

Response Fig. 1: Mcam/CD146 expression in LSECS. a. LSECs manifest the highest expression of Mcam/CD146 among different hepatic cell types. Shown in a reanalysis of the Tabula Muris liver dataset. **b.** CD45-CD31-CD146- events were backgated and plotted on an FSC-A/SSC-A graph. These events are very small sized and potentially debris.

COMMENT 2: Clarification of LSEC Numbers and FACS Analysis (Figure 2): The figure legend for Figure 2 should clearly describe what the dot plot represents. Are the LSEC numbers evaluated by FACS analysis reflective of sorted cell numbers using fluorescent FACS beads? How many sorted CD31/CD146-positive cells were used for the gene expression analysis presented in Figure 3c? Additionally, do the sorted cells remain viable in culture? Please include the scale values for the size bars in Figure 2c to improve clarity.

LSEC numbers are evaluated by FACS analysis (evaluated using fluorescent FACS beads) and are reflecting numbers of sorted cells? How many sorted CD31/CD146 positive cells have been used for gene expression analysis described in Figure 3c? Do the sorted cells survive in culture?

RESPONSE 2: Yes, the data in Figure 2 are LSEC numbers calculated using CountBright plus FACS counting

beads. For clarity, we have adapted the corresponding figure legend in the revised manuscript.

During revisions, we analyzed the viability of FACS-sorted LSECs. We found around 80% of FACS-sorted LSECs are viable, based on the exclusion of trypan blue dye and subsequent counting on hemocytometer. We also plated FACS-sorted LSECs in culture dishes, however, less than 10% cells properly adhered to the dish surface. We have discussed the limitation of dual (MACS+FACS) sorting approach in the revised manuscript.

For the gene expression analysis in Figure 3c, we isolated mRNA from 500,000 FACS-sorted LSECs. We have included this information in the methods part of the revised manuscript.

We apologize for missing the scale bar value. We have added this information in the revised manuscript.

<u>COMMENT 3:</u> Controversy Regarding CD31 as an LSEC Marker: CD31 expression in mouse LSECs has been a subject of debate, as it is sometimes associated with LSEC capillarization or endothelial cells from central veins. To address this, the authors should provide the percentage of Stabilin2-positive and Stabilin2-negative cells within the CD31/CD146 population, as determined by FACS analysis.

<u>RESPONSE 3:</u> We thank the reviewer for raising this critical point. We analyzed LSECs for Stabilin-2 and Lyve-1. For both markers, we observed a clear shift of positively stained population as compared to fluorescence minus one (FMO) control samples. These data are included in **Supplementary Figure S1** of the revised manuscript. When setting up positive gates based on corresponding FMO control samples for Stabilin-2 and Lyve-1 (**Response Fig. 2**), we observed ~98% of LSECs to be positive for Stabilin-2 and around 87% positive for Lyve-1, further validating the identity of our isolated LSECs.

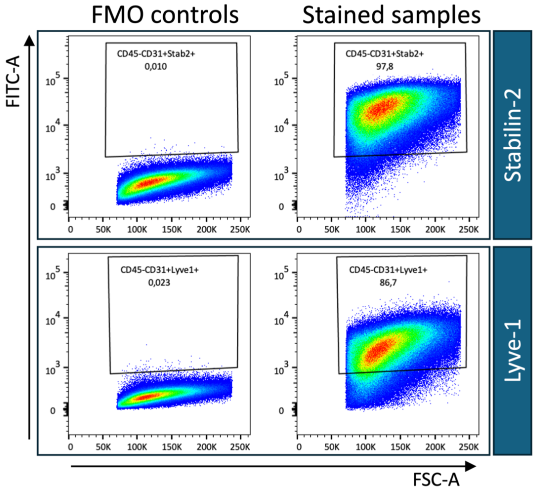

Response Fig. 2: Validating LSECs with Stabilin-2 and Lyve-1 staining. LSECs were stained and FACS analyzed for either Stabilin-2 or Lyve-1. For both markers, we observed a clear shift in positively stained population as compared to corresponding FMO control samples.

<u>COMMENT 4:</u> Heterogeneity of Cultured Cells (Figure 4a): In Figure 4a, the CD146-positive cells sorted by magnetic beads and stained with CD31 and CD32b do not exhibit membrane-specific staining, and the adherent cell population appears highly heterogeneous, as also seen in the FACS analysis and brightfield image. Has additional staining been performed to evaluate the presence of macrophages or stellate cells within the adherent population?

<u>RESPONSE 4:</u> We apologize for the poor quality of brightfield and immunofluorescence images in the first version of the manuscript. We now provide higher resolution images in the revised manuscript.

To systematically characterize our primary LSEC cultures, we first **stained our primary cultures with cell-specific markers** - CD31 and ERG (endothelial cell-specific), Desmin (stellate cell-specific) and F4/80 (macrophage-specific). These analyses revealed that our cultures primarily contain endothelial cells, with only 1-2 non-endothelial cells per field of view (corresponding to <2% of all DAPI+ cells). Secondly, we **characterize endothelial cells with sinusoidal markers** – Stab2 and CD32b. In concordance with the FACS analyses (see response to comment 3), these analyses suggest that the majority of cultured CD31+ endothelial cells are concurrently positive for CD32b and Stab2. Thirdly, we pursued **scanning electron microscopy** to show that our isolated endothelial cells are *bona fide* LSECs as they harbor fenestrations, a morphological hallmark of

LSECs. Together, we believe that these data (**Fig. 4** of the revised manuscript) comprehensively validate the purity and quality of our isolated LSECs.

COMMENT 5: Comparative analysis with perfusion-based isolation: One advantage of perfusion-based LSEC isolation followed by a density gradient for NPCs is the simultaneous isolation of both LSECs and macrophages. Did the authors evaluate the viability and number of macrophages within the CD146-negative (flow-through) population? Does this new digestion protocol enable efficient isolation of viable macrophages using anti-CD11b or F4/80 magnetic beads? This question is relevant given the suggestions in the discussion about the protocol's potential broader use.

RESPONSE 5: We thank the reviewer for this thoughtful suggestion. During revisions, we analyzed the presence of macrophages in the resulting NPCs after perfusion-independent digestion of liver tissues using our method. As shown in the **Supplementary Figure S3** of the revised manuscript, the resulting NPCs consist of viable CD45+F4/80+ macrophages (Kupffer cells). Based on our FACS analysis with counting beads, we could obtain approximately 1.5 x 10^6 F4/80+ macrophages per gram mouse liver tissue (**Fig. S3** of the revised manuscript). These numbers are consistent with a recent publication describing a perfusion-based method to isolate liver macrophages (5). These data suggest that our perfusion-independent method can be applied for simultaneous isolation of both LSECs and macrophages. We have discussed this further in the revised manuscript.

COMMENT 6: Use of CD117 as a marker for zonation: CD117 (c-Kit) is primarily known for its role in hematopoiesis, stem cell maintenance, and tissue regeneration and is more commonly associated with hepatic progenitor cells or stellate cells. Its use as a marker for LSEC zonation is less established.

Could the authors explain their rationale for using CD117 in this context? Also, the methodology for CD117-based stratification should be more detailed. Were different CD117-expressing populations sorted from a double-stained CD146/CD31 sorted population (is this the sequential approach?) The total number of CD117-high, CD117-mid, and CD117-low sorted cells should also be expressed per gram of liver tissue.

RESPONSE 6: We thank the reviewer for raising this point. We are well-aware of the fact that CD117 (c-Kit) is heavily expressed on hematopoietic stem and precursor cells. Yet, the single-cell datasets have recently shown that LSECs express similar levels of Kit to hematopoietic cells

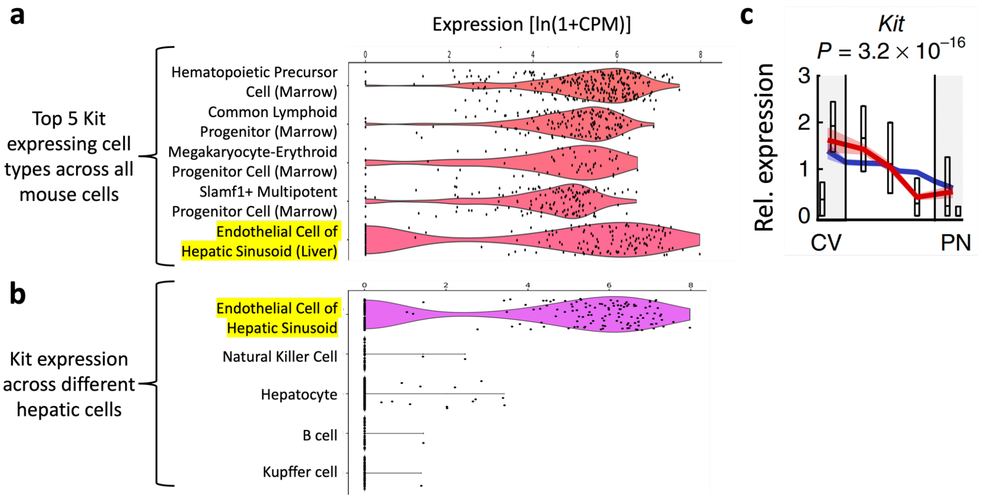

Response Fig. 3: LSECs express CD117 (c-Kit). a. Tabula Muris data set was reanalyzed to observe highest CD117 (c-Kit) expressing cells among all mouse cells. Five cell types with highest levels of Kit, include hematopoietic stem and precursor cells and liver sinusoidal endothelial cells. **b.** Analysis of Kit expression across different cell types in the liver revealed LSECs as hepatic cell type expressing highest levels of Kit. **c.** Shown is the Kit expression in LSECs along a liver sinusoid with higher levels of Kit expression in pericentral LSECs (Blue line – scRNAseq; Red line – FISH-based validation). Shown data are adapted from Halpern *et al. Nat. Biotech* 2018 (4).

(**Response Fig. 3a**). Moreover, within different liver cells, LSECs show the highest level of Kit expression (**Response Fig. 3b**). Interestingly, Kit expression in LSECs is zonated along the hepatic sinusoid as shown by Halpern *et al. Nat. Biotech* 2018 (**Response Fig. 3c**) (4), with pericentral LSECs having the highest Kit expression. Subsequently, Inverso *et al. Dev Cell* 2021 (6) exploited these findings to establish a FACS-based strategy to isolate LSECs from different hepatic zones, stratifying LSECs into pericentral, midlobular and periportal based on the surface abundance of Kit. In the current work, we employ this strategy to demonstrate that we can similarly stratify LSECs isolated with our perfusion-independent method into different zones of the liver sinusoid for downstream applications.

Technically, we employed a nested gating strategy where CD45-CD31+CD146+ cells were sequentially analyzed for the surface abundance of CD117 (c-Kit). In line with Inverso *et al. Dev Cell* 2021 (6), we defined 15% LSECs (~1.5 x 10^6 per g liver tissue) with the highest Kit staining as pericentral, lowest 15% LSECs (~1.5 x 10^6 per g liver tissue) as periportal and the rest intermediate expressing cells as midlobular LSECs. We have expanded in the revised manuscript.

COMMENT 7: Additionally, please specify the fluorescent-labeled antibodies used, not only the antibody clones.

RESPONSE 7: We have provided a table outlining all information relevant to used FACS antibodies in the revised manuscript.

COMMENT 8: Also, why wasn't LYVE1, a more traditional marker for LSEC zonation, used for stratification? What was the LYVE1 gene expression in the CD117-sorted cells, as shown in Figure 4d?

RESPONSE 8: As shown in the **Response Figure 4**, we observed no significant differences in the expression of Lyve1 when comparing pericentral (PC), midlobular (MD) and periportal (PP) LSECs.

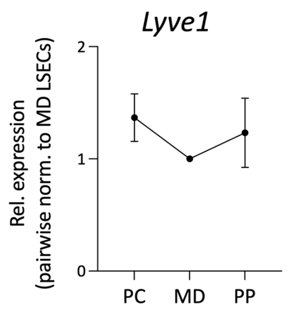

Response Fig. 4: Lyve1 expression in CD117-zonated LSECs. [mean ± SD, n = 4 mice]. There is no difference based on one-way ANOVA test.

COMMENT 9: In the introduction, the statement "The accumulation of excessive extracellular matrix requires longer perfusion times with digestion mix and negatively affects the yield of LSEC isolation" needs a reference.

RESPONSE 9: We have included a citation for the requirement of longer perfusion times for digesting fibrotic livers in the revised manuscript. Subsequent negative impact of longer digestion times on the LSEC yield is based on our own personal experiences and discussion with several liver research groups at Heidelberg University. For accuracy, we rephrased this statement in the revised manuscript.

Reviewer #2

GENERAL COMMENT: The authors present a perfusion-independent protocol for isolation and purification of mouse liver sinusoidal endothelial cells (LSECs), where they chop the liver tissue and digest the tissue with liberase solution followed by differential centrifugation to remove hepatocytes. They then use a combination of MACS (CD31+,CD146+), and FACS (sorting viable, CD45- CD31+ cells) for cell purification. Liberase is commonly used in perfusion-based methods for LSEC isolation, and known to be effective while at the same time gentle to the cells. Cell yield per gram liver in the presented method is comparable to established perfusion-dependent methods which represents an improvement compared to other perfusion-independent protocols for LSEC isolation. The method further allows isolation of LSECs from both normal and fibrotic liver samples. A simplified method was used for purification of pig LSECs.

I find the protocol well described and potentially very useful. My specific comments to the study are linked to the characterization of the purified cells.

RESPONSE TO GENERAL COMMENT: We sincerely thank the reviewer for his/her positive assessment and valuable feedback on our manuscript. A concerted effort was made to address all comments of the reviewer which – we believe – has advanced our manuscript further.

COMMENT 1: The endothelial markers CD31 and/or CD146 do not distinguish between LSEC and other endothelial cells in liver, although LSECs represent by far the largest population of liver endothelial cells. A morphological hallmark of LSECs is their numerous fenestrae, which are open nanosized holes with a diameter below the resolution limit of the light microscope (average diameter 100-200 nm). The purified endothelial cells from mouse and pig liver should therefore be characterized by electron microscopy or other super resolution imaging methods which allow for visualization of fenestrations, as part of the phenotyping of the cells in this method paper.

RESPONSE 1: We thank the reviewer for raising this critical point. During revisions, we pursued scanning electron microscopic analysis of our primary LSEC cultures. As shown in **Figure 4d** of the revised manuscript, primary LSECs isolated with our method harbored fenestrations. Further, we pursued analysis of additional sinusoidal markers, such as Stabilin-2 to further validate the identity of our isolated LSECs during revisions.

COMMENT 2: Have you observed any differences in plating efficiency, spreading and survival in culture of cells that has been through both MACS and FACS purification steps, versus MACS alone? This is of interest for the choice of purification method when performing functional studies in primary LSEC cultures.

RESPONSE 2: We thank the reviewer for this thoughtful comment. Comparing the culture efficiency of LSECs isolated with MACS+FACS versus MACS alone, we could observe a clear difference with LSECs isolated with MACS+FACS adhering in far fewer numbers (only around 10% of cells properly adhered and spread on the dish surface) as compared to MACS alone. Perhaps LSECs are too stressed when they are processed by FACS. We have discussed the limitations of the dual (MACS+FACS) sorting approach in the revised manuscript. Overall, we believe that MACS alone is sufficient to establish primary cultures while MACS+FACS is required for more sensitive downstream assays, such as RNAseq and proteomics, which require a very high purity of LSECs.

Reviewer #3

GENERAL COMMENT: This is an experimental study, describing a novel strategy to isolate liver sinusoidal endothelial cells (LSECs) from mice and pigs.

LSECs are notriously difficult to isolate and culture and therefore, any protocol to improve isolation and culture of LSECs is welcome.

The method described in this study is a significant improvement over existing methods, especially with respect to ease of isolation, time required and yield and will make it possible for many more groups to study this interesting, but difficult to obtain, cell type.

However, what is lacking are functional studies with the LSECs that are obtained, as well as a more detailed characterisation.

RESPONSE TO GENERAL COMMENT: We sincerely thank the reviewer for his/her positive assessment and valuable feedback on our manuscript. A concerted effort was made to address all comments of the reviewer which – we believe – has advanced our manuscript further.

COMMENT 1: How long can these LSECs be kept in culture in a differentiated state; does it differ from perfusion-dependent obtained LSCEs?

RESPONSE 1: We performed a sidewise isolation of LSECs with our perfusion-independent method and traditional perfusion-dependent liver digestion method. In principle, we observed comparable culture characteristics of LSECs isolated with either of the methods. Similar to LSECs isolated with the perfusion-dependent method, we could passage them with Accutase-based cell detachment.

We additionally collected samples for mRNA analysis at different time points after seeding LSECs. Here, we compared the temporal dynamics of gene expression of CD31 (Pecam1), Stabilin-2 and Lyve-1 in cultured LSECs between LSECs that were isolated using either our method or perfusion-dependent method. Overall, we observed very similar kinetics of gene expression for the analyzed markers between the two methods (**Response Fig. 5**) with no statistically significant differences between LSECs isolated with either of the approaches. Hence, we believe that cultured LSECs with our perfusion-independent method behave similarly to LSECs isolated with current perfusion-dependent liver digestion methods.

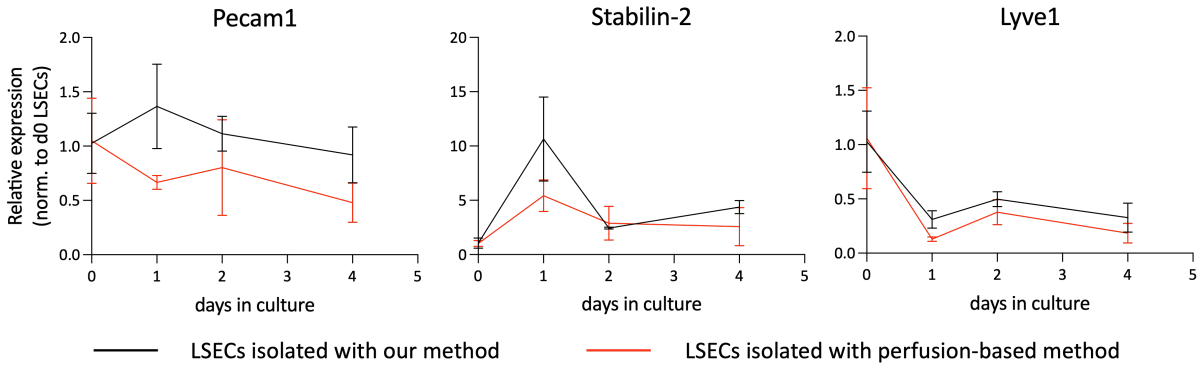

Response Fig. 5: Comparing temporal expression kinetics of selected genes between LSECs isolated with our perfusion-independent method and current perfusion-dependent liver digestion method. The comparisons were rendered non-significant between two groups by two-way ANOVA test.

COMMENT 2: Do these LSECs have fenestrae, confirmed by electron microscopy?

RESPONSE 2: We thank the reviewer for raising this critical point. During revisions, we performed scanning electron microscopy on our primary LSEC cultures. As shown in **Figure 4d** of the revised manuscript, primary LSECs isolated with our method harbored fenestrations.

COMMENT 3: The authors rely heavily on CD31 as marker for LSECs. LSECs have been reported to specifically express also other LSECs specific markers, e.g. LYVE-1, stabilins (as reported previously by this group), scavenger receptors, eNOS (not specific for LSECs, but a functional trait of all endothelial cells.

RESPONSE 3: While revising the manuscript, we pursued a systematic characterization of our isolated LSECs. Here, we performed FACS analysis with additional markers, including Lyve-1 and Stabilin-2 (**Figure S1a** of the revised manuscript) and immunofluorescence imaging with Stabilin-2 and CD32b (**Figure 4b,c** of the revised manuscript). Together, these new data, together with scanning electron microscopy analysis, substantiate the sinusoidal nature of isolated liver ECs.

COMMENT 4: How did the authors rule out the contamination of non-sinusoidal endothelial cells?

RESPONSE 4: We thank the reviewer for this thoughtful comment! During revisions, we characterize our primary cultures with different sinusoidal markers – Stab2 and CD32b (**Figure 4b,c** of the revised manuscript). We observed that the majority of cultured endothelial cells are concurrently positive for CD32b and Stab2. Yet, we agree with the reviewer that we cannot absolutely rule out a minor contamination of non-sinusoidal endothelial cells with the current analyses.

References:

1. Elvevold KH, Nedredal GI, Revhaug A, Smedsrod B. Scavenger properties of cultivated pig liver endothelial cells. Comp Hepatol **2004**;3:4
2. Nedredal GI, Elvevold KH, Ytrebo LM, Olsen R, Revhaug A, Smedsrod B. Liver sinusoidal endothelial cells represents an important blood clearance system in pigs. Comp Hepatol **2003**;2:1
3. Guo Q, Furuta K, Aly A, Ibrahim SH. Isolation and characterization of mouse primary liver sinusoidal endothelial cells. J Vis Exp **2021**
4. Halpern KB, Shenhav R, Massalha H, Toth B, Egozi A, Massasa EE*, et al.* Paired-cell sequencing enables spatial gene expression mapping of liver endothelial cells. Nat Biotechnol **2018**;36:962-70
5. Andreata F, Bleriot C, Di Lucia P, De Simone G, Fumagalli V, Ficht X*, et al.* Isolation of mouse Kupffer cells for phenotypic and functional studies. STAR Protoc **2021**;2:100831
6. Inverso D, Shi J, Lee KH, Jakab M, Ben-Moshe S, Kulkarni SR*, et al.* A spatial vascular transcriptomic, proteomic, and phosphoproteomic atlas unveils an angiocrine Tie-Wnt signaling axis in the liver. Dev Cell **2021**;56:1677-93 e10

Response to Reviewer's comments

Reviewer #1

The revised manuscript has been significantly improved, and my previous concerns and questions have been addressed thoroughly.

Reviewer #2

The authors have very nicely answered all my questions.

Reviewer #3

The authors significantly improved the quality of the manuscript and addressed all my comments and concerns in a satisfactory way.

RESPONSE: We sincerely thank all reviewers for taking the time to review our manuscript and appreciate their valuable and constructive feedback. Thank you!
